# Unveiling Artificial Intelligence’s Power: Precision, Personalization, and Progress in Rheumatology

**DOI:** 10.3390/jcm13216559

**Published:** 2024-10-31

**Authors:** Gianluca Mondillo, Simone Colosimo, Alessandra Perrotta, Vittoria Frattolillo, Maria Francesca Gicchino

**Affiliations:** Department of Woman, Child and of General and Specialized Surgery, AOU University of Campania “Luigi Vanvitelli”, Via Luigi De Crecchio 4, 80138 Naples, Italy; simone.colosimo@studenti.unicampania.it (S.C.); alessandra.perrotta@studenti.unicampania.it (A.P.); vittoria.frattolillo@studenti.unicampania.it (V.F.); mariafrancesca.gicchino@unicampania.it (M.F.G.)

**Keywords:** artificial intelligence (AI), rheumatology, diagnosis

## Abstract

This review examines the increasing use of artificial intelligence (AI) in rheumatology, focusing on its potential impact in key areas. AI, including machine learning (ML) and deep learning (DL), is revolutionizing diagnosis, treatment personalization, and prognosis prediction in rheumatologic diseases. Specifically, AI models based on convolutional neural networks (CNNs) demonstrate significant efficacy in analyzing medical images for disease classification and severity assessment. Predictive AI models also have the ability to forecast disease trajectories and treatment responses, enabling more informed clinical decisions. The role of wearable devices and mobile applications in continuous disease monitoring is discussed, although their effectiveness varies across studies. Despite existing challenges, such as data privacy concerns and issues of model generalizability, the compelling results highlight the transformative potential of AI in rheumatologic disease management. As AI technologies continue to evolve, further research will be essential to address these challenges and fully harness the potential of AI to improve patient outcomes in rheumatology.

## 1. Introduction

Artificial intelligence (AI) is gradually transforming the landscape of medical practice across various specialties. In fields such as radiology, oncology, and cardiology, AI-driven tools have already begun to increase diagnostic accuracy, optimize treatment strategies, and predict patient outcomes with unprecedented precision. These advancements provide a promising foundation for exploring the potential of AI in other complex areas of medicine, including rheumatology. By analyzing large datasets and identifying patterns that may not be immediately apparent to human clinicians, AI has the capacity to offer new perspectives on how chronic and multifaceted conditions are managed.

**Figure 1:**
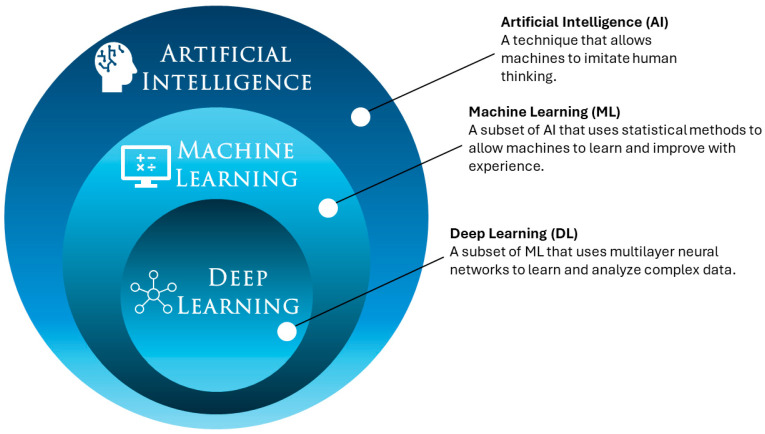
Graphical representation of various AI subcategories.

The increasing prevalence and complexity of rheumatoid arthritis (RA), a chronic autoimmune disease characterized by persistent inflammation, continue to pose challenges in effective management and treatment. Recent technological developments, particularly in AI, have opened new possibilities for improving the diagnosis, management, and treatment outcomes of rheumatologic diseases [1]. AI, which encompasses machine learning (ML) and deep learning (DL), refers to systems that can learn from data and perform that are traditionally associated with human intelligence. These technologies are gradually being integrated into various medical applications, ranging from diagnostics to personalized treatment plans (Figure 1) [2].

ML, a subset of AI, involves algorithms that can analyze and learn from data and make decisions without being explicitly programmed [3]. DL, a more complex subset of ML, uses neural networks with many layers, enabling the analysis of large datasets with a high degree of accuracy and the recognition of patterns that are not immediately apparent to humans [4]. Both AI techniques have shown potential in the development of diagnostic tools, predictive models, and personalized treatment strategies in medicine [5]. While the adoption of AI techniques in rheumatology is growing rapidly, as highlighted by García A.M. et al. [6], who reported a significant increase in annual scientific publications on the subject between 2017 and 2021 (from 20.2% to 48.3% compared with the previous year), the field is still in an exploratory phase. The applications of AI range from disease prediction and classification to research on both common and rare diseases, illustrating the growing interest of the scientific community in the potential applications of these technologies. However, it is important to recognize that AI in rheumatology is still a developing area, and further research is needed to demonstrate its clinical utility. Despite these advancements, significant gaps remain in understanding the full potential of AI in rheumatology. Large-scale studies evaluating the long-term effectiveness of AI-driven tools are still limited, particularly for pediatric populations and in the management of complex autoimmune diseases. Moreover, many current AI models rely on datasets that are not specifically tailored to rheumatology, limiting their generalizability across diverse patient groups and conditions. By harnessing AI capabilities, healthcare providers could offer more precise assessments, anticipate disease progression, and tailor treatments to individual patient needs, potentially improving overall patient outcomes in rheumatology. This review aims to critically assess the current applications of AI in rheumatology, highlight its potential, and address the limitations and challenges associated with its implementation, emphasizing the need for further research and comprehensive clinical studies to fully realize its potential in this field.

## 2. Roles of AI in Rheumatology

In this review, we explore the key applications of AI in rheumatology, focusing on four main areas: differential diagnosis, prognosis prediction, treatment personalization, and continuous monitoring. These domains represent emerging fields where AI has the potential to increase the accuracy and efficiency of patient care. Table 1 provides a summary of the cited studies, highlighting the most significant contributions to each of these areas.

### 2.1. Differential Diagnosis

Differential diagnosis is a crucial aspect of medical practice that benefits significantly from advancements in AI in the field of rheumatology. AI-driven methods offer a promising avenue for improving the precision of such diagnoses. This is exemplified in several studies that utilize convolutional neural networks (CNNs) and DL techniques to analyze medical imaging for better disease classification and prediction [37]. Venerito V. et al. used a ResNet34 CNN with transfer learning to assess the grade of synovitis from ultrasound-guided synovial tissue biopsies (USSBs). The model was trained on a dataset of 150 photomicrographs acquired at various magnifications (from 4× to 20×) and stained with hematoxylin and eosin (H&E). Each image was evaluated via Krenn’s score to differentiate between low- and high-grade synovitis. Transfer learning involves pretraining ResNet34 on the ImageNet database, followed by fine-tuning with the USSB dataset. Data augmentation techniques, such as rotation, zooming, and horizontal flipping, were applied to enhance the model’s robustness. The training dataset included 90 images (42 with high-grade synovitis), whereas the validation and test datasets each comprised 30 images. The model achieved 100% accuracy, precision, and recall in the test phase. To better understand the model’s decision-making process, the gradient-weighted class activation mapping (Grad-CAM) algorithm was employed. Grad-CAM is a visualization method that highlights the regions of an image that the model focuses on when making its decision. In this case, the algorithm generated a heatmap showing that the model concentrated primarily on the cellularity in the synovial lining and sublining layers, which are key determinants of Krenn’s score. This confirmed that the model focused on relevant features for classification, providing a visual explanation and further validation of the model’s correct functioning. In other words, Grad-CAM not only improved the interpretability of the model’s decision-making process but also helped verify that the predictions were based on clinically relevant pathological criteria. This finding demonstrates the potential of AI to significantly increase the accuracy of differential diagnoses in rheumatology [7]. Bonnin M. et al. [8] focused on RA, utilizing AI to automate the scoring of hand X-rays on the basis of the Sharp/van der Heijde (SvH) method [38]. This method involves a database of 3818 hand X-rays processed to classify patients and predict disease severity. The AI models trained on these data achieved high levels of prediction accuracy and classification, with a Pearson correlation of 0.86, a mean area under the curve (AUC) of 0.97, and an overall accuracy of 84%. Wang H-J. et al. proposed an advanced system utilizing YOLO (You Only Look Once), a DP model for the detection of joint abnormalities in hand X-ray images to assist in the diagnosis of rheumatoid arthritis (RA). Specifically, the system addressed challenges in detecting carpal bones and regions with joint space narrowing (JSN), which is crucial for the assessment of RA severity on the basis of the modified total Sharp/van der Heijde Score (mTSS). The YOLO model was chosen for its efficiency in object detection and its ability to process X-ray images in real time. In their study, Wang applied image preprocessing techniques, such as adjusting the window level to standardize the images, enhancing key features such as bone and joint structures, and reducing noise, which improved the accuracy of joint detection. They also employed data augmentation strategies such as image rotation and flipping to improve the model’s robustness. Their model achieved a mean average precision (mAP) of 0.92 for detecting joint space narrowing, a precision of 0.95, and a recall of 0.94. For the classification of joints on the basis of mTSS severity (healthy, mild, severe), the model obtained an average accuracy of 0.88, with accuracies of 0.91 for severe cases, 0.79 for mild cases, and 0.90 for healthy joints. One notable finding of their research was the difficulty in detecting intercarpal joints due to their complex structure compared to finger joints. However, despite this challenge, the YOLO-based system outperforms traditional methods and has shown potential for use in clinical settings by significantly reducing the time required for the manual scoring of X-rays, thus improving diagnostic efficiency. The study further emphasized the importance of explainable AI, which uses methods such as Grad-CAM to highlight the regions in the X-rays that the model focused on when making its predictions. This not only improved the transparency of the model’s decision-making process but also built trust among clinicians, who could verify that the AI was making decisions on the basis of medically relevant features [9]. Xu et al.’s meta-analysis demonstrated that AI models used for diagnosing temporomandibular joint osteoarthritis (TMJOA) through radiographic imaging have high sensitivity (80%) and specificity (90%), with an AUC of 92%. TMJOA, a degenerative condition often linked to rheumatoid arthritis (RA), results in cartilage deterioration and joint pain. In RA patients, systemic inflammation can lead to joint degeneration in the temporomandibular region, manifesting as limited jaw movement and pain. AI models have proven effective in detecting subtle structural changes in the joint, such as joint space narrowing and bone erosion, offering a more accurate and early diagnosis than traditional methods do. This capability is crucial for RA patients, where early intervention can prevent further joint damage and improve outcomes [10]. Further research has explored the use of extremity MR scans with AI to predict the onset of rheumatoid arthritis (RA) in its early stages and in patients with clinically suspected arthralgia. The study developed a predictive model using self-supervised DL to analyze scans from 1974 patients, which included 1247 individuals with early-onset arthritis and 727 with clinically suspected arthralgia. Self-supervised DL models were employed for image processing and predictive analysis. The AI model demonstrated strong predictive accuracy, achieving a mean AUC of 0.683 for early arthritis patients and 0.727 for arthralgia patients. A detailed analysis of specific joint areas further highlighted the model’s accuracy, with AUCs of 0.679 for the wrist, 0.647 for the metacarpophalangeal joints (MCPs), and 0.664 for the feet in early arthritis patients. In patients with arthralgia, the model achieved AUCs of 0.688 for the wrist, 0.669 for the MCPs, and 0.715 for the feet, underscoring its effectiveness in early detection and detailed disease profiling [11]. Van Leeuwen J.R.et al. [12] developed a natural language processing (NLP) model to improve patient identification for clinical research on ANCA-associated vasculitis, utilizing advanced AI methods, particularly text mining combined with NLP. In two medical centers, the AI tool demonstrated high sensitivity in identifying patients but initially had modest positive predictive values (PPVs). The incorporation of NLP-based exclusion refined the results, reducing the number of identified patients while significantly improving PPVs while maintaining high sensitivity. In the academic center, the PPV increased from 56.9% to 77.9%, and in the validation center, it increased from 58.2% to 86.1%. The sensitivity remained consistently high across settings. This highlights the effectiveness of NLP in increasing diagnostic precision and the generalizability of the AI tool across diverse healthcare systems. Burlina P. et al. [13] evaluated the effectiveness of a CNN algorithm in diagnosing myositis from muscle ultrasound images. The study utilized 3214 images from 80 subjects, including 19 with inclusion body myositis (IBM), 14 with polymyositis (PM), 14 with dermatomyositis (DM), and 33 healthy controls. The research focused on three key classification tasks: distinguishing between healthy controls and all types of myositis, differentiating IBM from other types of myositis (PM and DM), and evaluating the algorithm’s overall accuracy. In distinguishing healthy controls from myositis patients, the algorithm achieved an accuracy of 76.2 ± 3.1%. When differentiating IBM from the other types of myositis (PM and DM), the algorithm demonstrated an accuracy of 74.8 ± 3.9%, outperforming a machine learning Random Forest algorithm, which achieved an accuracy of 68.9 ± 2.5%.

### 2.2. Prognosis Prediction

In the field of rheumatology, the integration of AI for prognosis prediction has become increasingly notable, as evidenced by recent scholarly contributions. These works explored the ability of AI to analyze extensive medical datasets, offer improved disease management strategies, and even automate aspects of medical documentation and reporting. Wang J. et al. [14] reviewed the achievements that AI has made in the early detection and management of RA. Their survey underscored the pivotal role of AI in facilitating early diagnosis and timely intervention, which is crucial for halting the progression of chronic RA. However, they also noted the ongoing challenges, including the need for additional research to verify the precision of AI applications in this field while also considering the technological and ethical implications. Salmi J. et al. [15] specifically investigated how AI could leverage demographic and clinical data to predict the disease course in patients with RA. Using a ML algorithm, researchers aimed to predict the Disease Activity Score-28 for Rheumatoid Arthritis with CRP (DAS28-CRP) one year into the future. The study analyzed a dataset of 1881 RA patients, both seropositive and seronegative, to develop an AI-based approach for forecasting disease activity. The ML algorithm used a voting ensemble method combining Random Forest and Extreme Random Trees classifiers. The features included 30 clinical and demographic variables, such as age, sex, disease duration, and baseline clinical markers. The model achieved an AUC of 0.71 in predicting DAS28-CRP, offering valuable insights for clinicians in making more informed treatment decisions. Specifically, the model could guide adjustments in treatment plans by predicting whether a patient’s condition would worsen, allowing for earlier interventions such as increasing treatment intensity or scheduling more frequent follow-up visits. Conversely, if the model predicted improvement, it could suggest reducing the frequency of clinical visits. The most critical variables influencing the model’s predictions included baseline CRP (C-Reactive Protein) levels, age, and disease duration. The AI model’s ability to classify patients into different activity levels—ranging from inactive to very high disease activity—was particularly effective in identifying patients with high or very high activity. However, the model’s performance was less accurate in distinguishing between adjacent disease activity levels, such as between low and inactive disease. Despite these limitations, the study highlighted AI’s potential as a supportive tool for clinicians, providing insights that could lead to more personalized treatment strategies. Verhoeven F. et al. [16] shifted the focus to the role of AI in medical documentation and the literature. Their study discussed how AI platforms such as ChatGPT could not only assist in writing scientific papers but also extend to prognostic analytics in clinical settings. They highlighted the importance of human oversight to effectively manage the ethical complexities associated with AI applications. Al Shareedah A. et al. [17] delved into CatBoost, a ML algorithm, to predict Systemic Lupus Erythematosus (SLE) within an Omani cohort. An analysis of data from 219 patients, including 138 with SLE, revealed that the model demonstrated high predictive accuracy, reflected by an area under the receiver operating characteristic curve (AUROC) of 0.956, and exhibited a strong sensitivity of 92%. Through SHAP analysis, four key clinical features (alopecia, renal disorders, acute cutaneous lupus, and hemolytic anemia), alongside patient age, emerged as highly influential in terms of prediction accuracy. Yang et al. [18] aimed to develop a diagnostic model for primary Sjögren’s syndrome (pSS) by combining bioinformatics analysis and ML algorithms. Their approach involved identifying 96 differentially expressed genes (DEGs) from publicly available datasets. Subsequently, a Random Forest classifier was employed to select 14 key genes, which were then used to construct diagnostic models based on artificial neural networks (ANN), Random Forest, and Support Vector Machines (SVM). The models achieved excellent diagnostic performance with AUC values of 0.972, 1.00, and 0.9742 in the training set, respectively. In the validation set, the Random Forest model showed the best predictive performance with an AUC of 0.8321, suggesting its potential utility in the clinical screening of pSS. An adaptive DL algorithm, AdaptiveNet [19], was developed to predict the Disease Activity Score in 28 joints (DAS28) in rheumatoid arthritis (RA) patients via demographic and clinical data from the Swiss Clinical Quality Management Registry. The model utilized Long Short-Term Memory networks (LSTM) to process patient histories and was evaluated with 5-fold cross-validation, achieving an AUC of 0.73. The key predictors included painful joints, disease duration, and age, demonstrating the model’s potential to enhance personalized RA management.

Norgeot B. et al. [20] developed a neural network model using DL techniques to predict disease activity in RA patients, specifically focusing on the Clinical Disease Activity Index (CDAI). The model was built using electronic health record (EHR) data, including patient demographics, medications, and laboratory values, such as C-reactive protein (CRP) and the erythrocyte sedimentation rate (ESR). This DL approach achieved a remarkable AUROC of 0.91 when predicting the CDAI at the next clinical visit for RA patients treated at a university hospital. The model outperformed traditional methods, such as the use of a patient’s most recent CDAI score, which has a random predictive performance. This AI model provides a significant advancement in predicting disease progression and helps to inform therapeutic decisions by offering personalized predictions on the basis of individual patient histories and clinical data.

Wei et al. [21] further demonstrated the efficacy of ML algorithms in predicting the incidence of coronary heart disease (CHD) in RA patients, a population already at increased cardiovascular risk. This present study developed and validated a nomogram using demographic, clinical, and serological data, including age, sex, hypertension status, anti-cyclic citrullinated peptide (anti-CCP) antibody status, and lipid profiles. The model achieved an AUC of 0.77, outperforming traditional clinical scores such as the Framingham Risk Score in assessing cardiovascular risk in RA patients. This ML approach not only enhances the accuracy of CHD prediction but also emphasizes the importance of incorporating RA-specific risk factors such as inflammation and the immune response, which are often overlooked by conventional models.

A study conducted by Konstantonis G. et al. [22] developed a ML model for detecting cardiovascular disease (CVD) in 542 individuals with rheumatoid arthritis, diabetes, and/or hypertension. Using conventional biomarkers, blood tests, and ultrasound images, the model applied Random Forest, SVM, and Linear Discriminant Analysis. With data augmented by the SMOTE strategy, the model achieved an AUC of 0.98 and significantly outperformed traditional CVD risk scores, highlighting its effectiveness in predicting CVD in high-risk populations.

### 2.3. Treatment Personalization

The application of AI in rheumatology has significantly enhanced the personalization of treatment strategies, as highlighted in several recent studies that focused on the integration of ML and DP algorithms. These technologies not only revolutionized diagnostic processes but also facilitated tailored therapeutic approaches that directly impacted patient care. Bonnin M. et al. [8] discussed how automating the SvH scoring system with AI helped in making more personalized treatment decisions on the basis of quantified lesion severity. The classification algorithm achieved a mean AUC of 0.97, with an accuracy of 0.84 and a positive predictive value of 0.91. This application accelerated the diagnostic imaging process, reduced subjectivity in scoring, and ensured that therapeutic strategies were better tailored to individual patient needs. Moreover, the development of ATRPred, a machine learning-based tool, underscored AI’s ability to predict treatment responses. This tool forecasted anti-TNF treatment response in RA patients with an accuracy of 81%, sensitivity of 75%, and specificity of 86% [23]. These results highlighted ATRPred’s effectiveness in identifying RA patients likely to benefit from anti-TNF therapies, potentially leading to improved treatment outcomes and cost reductions associated with ineffective treatments. Westerlind H. et al. [24] conducted a study aimed at predicting the persistence of methotrexate (MTX) therapy in patients with RA via ML algorithms. Their model incorporated demographic and clinical data, including patients’ medical history and laboratory data, to predict the likelihood of continuing MTX therapy for at least one year. The study included 5475 RA patients, 70% of whom remained on MTX monotherapy after one year. The researchers used multiple ML models, including Lasso regression, Random Forest, and SVM, and tested their performance via the AUROC. The highest-performing model, Lasso regression, achieved an AUROC of 0.67, whereas traditional hypothesis-based methods achieved an AUROC of 0.66, confirming the superiority of ML approaches for this prediction task, albeit with a modest improvement. The study highlighted that clinical factors at diagnosis, such as disease activity and age, were the most important predictors for persistence on MTX. Surendran S. et al. [25] developed a ML model that demonstrated significant efficacy in predicting liver enzyme elevation, achieving an F1 score of 0.87. The model revealed that baseline transaminase levels, along with lymphocyte and neutrophil counts, were the most influential parameters in predicting an increase in transaminase levels. Morid M. et al. [26] evaluated various ML techniques to predict the need for a treatment step-up within one year among RA patients. The one-class SVM demonstrated the best performance, with a recall of 89% and a precision of 51%, achieving an F-measure of 65%. Artacho A. et al. [27] developed a Random Forest algorithm to predict the MTX response in patients with RA on the basis of gut microbiome data. This study analyzed the microbiome profiles of several RA patients, aiming to identify specific characteristics associated with a positive response to MTX treatment. The model achieved an AUC of 0.84, demonstrating a good ability to distinguish between responders and non-responders to treatment. When the model focused exclusively on patients with either a high (≥80%) or low (≤20%) probability of response, the AUC increased to 0.94, suggesting that the model is particularly effective at identifying patients at the extremes of response likelihood. This improvement in the model performance indicated that the gut microbiome could provide valuable information for predicting treatment outcomes. A notable aspect of the study was the exclusion of pharmacogenetic predictors, highlighting that the gut microbiome itself was a strong predictor of the MTX response. These findings suggest that the biological mechanisms associated with the intestinal microbial composition play a key role in determining treatment efficacy. This innovative approach could support personalized medicine by improving the ability to identify patients who are most likely to benefit from MTX treatment, reducing the use of ineffective treatments, and improving overall clinical outcomes. ML algorithms have also been effective in predicting the response to second- or third-line biological DMARDs (bDMARDs) via genomics data. For example, Kim et al. developed an SVM model to predict the response to infliximab in RA patients on the basis of synovial tissue gene expression profiles. This study utilized differentially expressed genes (DEGs) as input features and identified key biomarkers associated with treatment response. The SVM model achieved an impressive AUC of 0.92 and an area under the precision–recall curve (AUPR) of 0.86, demonstrating high predictive accuracy. This model was particularly effective in distinguishing between responders and non-responders to infliximab, a TNF-α inhibitor commonly used as a second- or third-line therapy in patients who have not responded to conventional DMARDs. By incorporating genomics data, the algorithm was able to capture the underlying biological processes driving drug efficacy, offering a more precise approach to personalizing RA treatment. Additionally, the study highlighted the potential of using synovial tissue-specific gene expression profiles, rather than peripheral blood data, to improve prediction models for RA drug responses [28]. Kato M. et al. [29] developed a scoring system utilizing imaging data, specifically ultrasound images of synovitis, tenosynovitis, and enthesitis, in patients with RA and spondyloarthritis to predict treatment response. Their approach incorporated an unsupervised Random Forest algorithm along with uniform manifold approximation and projection to divide patients into two clusters on the basis of treatment response, as measured by the American College of Rheumatology 20, 50, and 70 criteria (ACR20/50/70). This method revealed distinct responses to treatment between the identified clusters, demonstrating the potential of imaging data in predicting treatment outcomes. Knitza J. et al. [30] presented a significant advancement in the field by integrating ML algorithms into the Rheport system [39]. The objective was to increase diagnostic accuracy, which was previously limited by an expert-driven weighted sum score system. By utilizing data from a national rheumatology registry of 2265 patients, 30.5% of whom were diagnosed with an Inflammatory Rheumatic Disease (IRD) and predominantly female (69.3%), nine ML algorithms were tested. All the models outperformed the existing algorithms, with new model AUROCs ranging between 0.630 and 0.737, significantly improving upon the AUROC of the current Rheport algorithm of 0.534. Specifically, the logistic regression model markedly increased the specificity from 17% to 33% while maintaining a 90% sensitivity level. The key features influencing model performance included finger joint pain, inflammatory marker levels, psoriasis, symptom duration, and sex. This integration of ML into Rheport not only enhances diagnostic accuracy but also promises more efficient patient triage, leading to timely interventions and improved outcomes [30].

### 2.4. Continuous Monitoring

Continuous monitoring in rheumatology has led to significant advancements through the integration of digital health technologies and ML, as demonstrated in several recent studies. Creagh A.P. et al. [31] explored the use of wearable devices and mobile applications to enhance the monitoring and management of rheumatoid arthritis. The use of wearable devices and ML algorithms significantly improved the monitoring of rheumatoid arthritis. The collected data enabled a more accurate distinction between patients and healthy individuals (F1 score 0.807). The integration of sensor data and patient-reported outcomes (PRO) improved the ability to stratify disease severity by increasing the F1 score to 0.833, making monitoring more precise than using PROs alone. Labat G. et al. [32] reported that, over the 36-week study period, the use of wearable activity trackers did not significantly reduce the number of flare episodes compared with the control group, as both groups presented similar flare rates (*p* > 0.05 at all time points). However, the study did find a modest improvement in daily physical activity levels, with an increase in step count among the tracker group, although this did not directly correlate with a reduction in flare episodes. Additionally, Hamy V. et al. [33] reported that the use of a smartwatch and a mobile application on a smartphone improved the accuracy of symptom data collection in patients with RA through real-time symptom recording. The authors demonstrated that these digital technologies were able to effectively monitor the daily impact of the disease on patients’ physical function, clearly distinguishing between RA patients and healthy controls. Data collection was reproducible over time and allowed for the continuous assessment of symptoms such as stiffness, pain, and fatigue. Compared with traditional periodic evaluations, this approach could enhance clinical management, enabling more detailed and personalized disease monitoring. Davergne T. et al. [34], in a review, discussed the usefulness of wearable devices for monitoring disease activity. Previous studies have shown that these devices can passively collect physical activity data, achieving accuracy rates above 95% in predicting disease flares in rheumatic conditions through the application of ML. However, they also noted concerns regarding the lack of common validation protocols and discrepancies between different devices. Gossec L. et al. [35] developed a Naïve Bayes model to detect flares in RA patients via physical activity data collected from wearable devices. The study was part of the ActConnect project, which involved 155 patients (82 with RA) who wore consumer-grade activity trackers to monitor steps per minute over a three-month period. Flares were self-reported weekly, and the data from the activity trackers were used to build a ML model capable of predicting these flare-ups. The model achieved high predictive performance, with a sensitivity of 95.7% and a specificity of 96.7% in detecting patient-reported flares. This accuracy was made possible through the use of continuous activity monitoring, which allowed the model to detect significant changes in physical activity patterns that corresponded to flare events. For example, during weeks with flares, patients showed a reduction in physical activity, with a 12–21% decrease in steps per day. This translates to an average decrease of 836 to 1462 steps per day, further confirming the association between flares and reduced physical activity.

Cobb R. et al. [36] presented detailed findings on the application of AI for continuous monitoring in RA via innovative imaging techniques. Their study involved 48 patients and 192 ⁹⁹mTc-maraciclatide scans, which were analyzed to segment regions of normal, low, and highly inflamed tissue. The nnU-Net model consistently outperformed the thresholding model, with higher modified Dice scores: 0.94 ± 0.01 for normal tissue, 0.51 ± 0.14 for low inflammation, and 0.76 ± 0.16 for high inflammation. The innovative use of ⁹⁹mTc-maraciclatide scans with AI models such as nnU-Net (no-new-Net) significantly contributed to the continuous monitoring of disease progression in patients with RA, enabling real-time patient management.

## 3. Discussion

The integration of AI into rheumatology has led to a significant evolution in the field, particularly in enhancing diagnosis, personalizing treatment, and enabling continuous monitoring. AI technologies, including ML and DL, have demonstrated considerable potential in improving diagnostic accuracy, predicting disease trajectories, and optimizing patient care. These advancements have been especially impactful in managing complex rheumatologic conditions such as RA and SLE. One of the most promising AI applications in rheumatology involves diffusion models, which represent a sophisticated approach to medical imaging. These models work by iteratively refining images through the controlled addition and removal of noise, allowing for the generation of highly realistic medical images. This technology was particularly valuable in scenarios where converting images from one modality to another—such as from CT to MRI—enhanced diagnostic precision. For example, the ability to generate MRI-like images from CT scans significantly reduces the need for multiple imaging procedures, saving both time and costs while minimizing the patient’s exposure to radiation. Conversely, converting MRI to CT provides essential anatomical details without subjecting patients to additional radiation, further improving the safety and efficiency of rheumatologic care [40]. In addition to imaging, AI has shown great promise in prognosis prediction. By analyzing extensive datasets, AI models were able to forecast disease progression and treatment responses with impressive accuracy. These predictive capabilities are crucial for conditions such as RA, where early and precise intervention could profoundly impact patient outcomes. The predictive models discussed in this review offer a quantitative assessment of disease activity, helping clinicians anticipate potential flares and adjust treatment plans preemptively [41]. The role of AI in personalizing treatment is another area of substantial progress. AI models leverage patient-specific data to recommend tailored therapies, enhancing efficacy and reducing adverse effects. This approach not only improved patient satisfaction but also improved long-term outcomes. However, it is essential to acknowledge that while AI has tremendous potential, it is not a substitute for clinical judgment. AI tools were designed to complement the expertise of healthcare professionals, providing them with enhanced data and insights that support decision-making processes. The human element remains crucial in interpreting AI-generated data, ensuring that patient care remains holistic and individualized. In pediatric rheumatology, there is a notable lack of studies focused on evaluating rheumatologic conditions in children, despite the importance of early diagnosis for better outcomes. A recent study analyzed 511 pediatric echocardiograms using DL to detect rheumatic heart disease (RHD) and focused on mitral regurgitation jets. The AI model effectively localized the left atrium with 99% precision and identified the correct systolic frame with over 93% accuracy, comparable to expert manual measurements. The model also showed strong diagnostic performance, with an AUC of 0.84, a precision of 78%, and a recall of 98% [42]. These applications and future perspectives demonstrate the broad potential of AI in rheumatology, highlighting how this technology is transforming the way rheumatologic diseases are diagnosed, managed, and treated. Table 2 provides a detailed overview of the main areas of AI application in rheumatology, illustrating current implementations and future development opportunities, with a particular focus on differential diagnosis, disease progression prediction, treatment personalization, and continuous monitoring.

## 4. Conclusions

In conclusion, the integration of AI into rheumatology represents a significant shift toward a more precise, data-driven, and patient-centered approach. AI has shown immense potential in enhancing various aspects of rheumatologic disease management, from early and personalized diagnosis to predicting treatment responses and continuously monitoring disease activity. These tools can help physicians make more informed decisions and tailor therapies to the specific needs of each patient. However, to fully leverage these technologies, issues related to data privacy, the development of standardized clinical protocols, and the adaptability of AI models to diverse patient populations must be addressed. It is essential that AI models are tested on an international scale to ensure their validity and reliability across different clinical settings. Moreover, further long-term studies are needed to evaluate not only the benefits but also the potential risks associated with the use of AI in rheumatology, particularly in pediatric populations, where diagnostic accuracy and timely intervention can make a significant difference. By maintaining a focus on these aspects, AI has the potential to revolutionize rheumatologic care, improve treatment efficacy, optimize clinical outcomes, and make the entire healthcare system more efficient and personalized.

## Figures and Tables

**Table 1:** AI applications in rheumatology. Summary of studies showcasing AI applications in diagnosing, predicting outcomes, personalizing treatments, and monitoring rheumatologic diseases.

Section	Authors	Results	Reference
Differential diagnosis	Venerito V. et al.	Utilized a ResNet34 CNN (Convolute Neural Network) with transfer learning to assess the grade of synovitis from ultrasound-guided synovial tissue biopsies. The model achieved 100% accuracy, precision, and recall in the test phase. Grad-CAM was employed to generate a heat map that confirmed the model’s focus on clinically relevant features.	[7]
	Bonnin M. et al.	Implemented an automated system for scoring hand X-rays using the Sharp/van der Heijde (SvH) method. Achieved 84% accuracy, with a Pearson correlation of 0.86 and an AUC of 0.97.	[8]
	Wang H-J. et al.	Developed a YOLO (You Only Look Once) model for detecting joint abnormalities in hand X-rays. Achieved a mean average precision of 0.92 and 0.88 accuracy in severe rheumatoid arthritis cases.	[9]
	Xu et al.	Meta-analysis showed AI’s effectiveness in diagnosing TMJOA (temporomandibular joint osteoarthritis) with 80% sensitivity, 90% specificity, and an AUC of 92%.	[10]
	Li Y. et al.	AI model using MRI scans to predict early RA and clinically suspect arthralgia, with an AUC of 0.683 for early arthritis and 0.727 for arthralgia.	[11]
	Van Leeuwen J.R. et al.	NLP model for improving patient identification in ANCA-associated vasculitis research, increasing PPV from 56.9% to 77.9%.	[12]
	Burlina P. et al.	CNN model for diagnosing myositis from ultrasound images with an accuracy of 76.2 ± 3.1%.	[13]
Prognosis prediction	Wang J. et al.	Reviewed AI’s impact on early RA diagnosis and management, highlighting AI’s role in early intervention.	[14]
	Salmi J. et al.	Developed a model to predict DAS28-CRP (Disease Activity Score-28 for Rheumatoid Arthritis with CRP) one year ahead in RA patients, achieving an AUC of 0.71.	[15]
	Verhoeven F. et al.	Explored AI’s role in scientific writing and predictive analytics for RA prognosis.	[16]
	Al Shareedah A. et al.	Machine learning model for predicting systemic lupus erythematosus (SLE) with an AUROC of 0.956 and 92% sensitivity.	[17]
	Yang K. et al.	Machine learning-based diagnostic model for primary Sjögren’s syndrome using 14 signature genes, with an AUC of 0.972 (ANN), 1.00 (RF), and 0.9742 (SVM) in the training set, and 0.766 (ANN), 0.8321 (RF), and 0.8223 (SVM) in the validation set.	[18]
	Kalweit M. et al.	AdaptiveNet deep learning model for predicting DAS28 in RA patients, achieving an AUC of 0.73.	[19]
	Norgeot B. et al.	Neural network model using EHR data to predict CDAI in RA patients, achieving an AUROC of 0.91.	[20]
	Wei T. et al.	ML nomogram for predicting CHD (coronary heart disease) in RA patients, achieving an AUC of 0.77.	[21]
	Konstantonis G. et al.	ML model for detecting cardiovascular disease in high-risk RA patients, with an AUC of 0.98.	[22]
Treatment personalization	Prasad B. et al.	Developed ATRPred tool for predicting anti-TNF treatment response in RA patients, with 81% accuracy, 75% sensitivity, and 86% specificity.	[23]
	Westerlind H. et al.	ML model to predict methotrexate therapy persistence in RA patients, achieving an AUROC of 0.67.	[24]
	Surendran S. et al.	ML model for predicting liver enzyme elevation in RA patients on methotrexate, achieving an F1 score of 0.87.	[25]
	Morid M. et al.	One-class SVM (Support Vector Machine) model to predict step-up therapy in RA patients, achieving 89% recall and 51% precision.	[26]
	Artacho A. et al.	Random Forest model to predict methotrexate response in RA patients using gut microbiome data, achieving an AUC of 0.84.	[27]
	Kim KJ. et al.	SVM model for predicting infliximab response using synovial tissue gene expression profiles, achieving an AUC of 0.92.	[28]
	Kato M. et al.	Developed a scoring system using ultrasound images to predict treatment response in RA and spondyloarthritis patients.	[29]
	Knitza J. et al.	ML improved diagnostic accuracy in the Rheport system, with AUROCs between 0.630 and 0.737.	[30]
Continuous Monitoring	Creagh A.P. et al.	Wearable devices and apps improved continuous RA monitoring, achieving an F1 score of 0.807.	[31]
	Labat G. et al.	Activity tracker study showed a 10% increase in physical activity but no significant reduction in flare episodes.	[32]
	Hamy V. et al.	Smartwatches and smartphone apps improved real-time symptom reporting in RA patients, allowing for faster clinical response.	[33]
	Davergne T. et al.	Wearable devices improved monitoring efficacy and accuracy for rheumatic disease activity.	[34]
	Gossec L. et al.	Naïve Bayes model detected flare-ups in RA patients using wearable devices, with 95.7% sensitivity and 96.7% specificity.	[35]
	Cobb R. et al.	AI segmentation of inflammation using ⁹⁹mTc-maraciclatide scans improved RA disease progression monitoring.	[36]

**Table 2:** Current and future AI applications in rheumatology. This table outlines the present uses of AI in rheumatology and highlights potential future developments across key areas such as differential diagnosis, disease progression prediction, treatment personalization, continuous monitoring, and research support.

Application Area	Current Use of AI	Future Perspectives
Differential Diagnosis	- Deep Learning for Medical Imaging: using convolutional neural networks (CNN) for automated analysis of ultrasounds, X-rays, and MRIs to identify rheumatologic conditions such as rheumatoid arthritis (RA), osteoarthritis (OA), and Sjögren’s syndrome with high accuracy. - Natural Language Processing (NLP): analyzing clinical notes to extract relevant information and support differential diagnosis. - Advanced Classification: assessing the severity of inflammation and identifying specific signs like bone erosions.	- Integrated Multimodal Models: developing algorithms that combine clinical data, imaging, and genetic information for earlier and more accurate diagnosis. - Explainability and Transparency: implementing explainable AI techniques to make decision-making processes more understandable and acceptable to clinicians. - Predictive Diagnosis: using AI to identify patients at risk of developing specific rheumatologic conditions early on.
Prognosis and Disease Progression Prediction	- Clinical Predictive Models: utilizing machine learning algorithms to estimate disease activity (e.g., DAS28-CRP) and the risk of cardiovascular complications in RA patients. - Therapeutic Response Prediction: analyzing data to anticipate treatment responses in different populations of patients.	- Integration of Omics Data: combining genomics, proteomics, and metabolomics with clinical data to create highly personalized and robust predictive models. - Unsupervised Learning: identifying clinical phenotypes and patient subgroups with similar prognoses, facilitating targeted therapeutic approaches. - Evolving AI Models: developing models that dynamically update with new clinical data, continuously improving prediction accuracy.
Treatment Personalization	- Drug Response Prediction: using clinical and genomic data to predict the effectiveness of treatments such as methotrexate or anti-TNF in RA patients. - Therapeutic Optimization: identifying patients who need therapy intensification or modifications to their current treatment.	- Advanced Multi-Omics Models: developing algorithms that integrate multi-omics data to create highly personalized treatment plans. - Side Effect Prevention Tools: creating predictive models to identify severe side effects and treatment-related complications early, improving therapeutic safety. - Precision Therapies: implementing treatment strategies based on individual molecular profiles, increasing effectiveness, and reducing unwanted effects.
Continuous Monitoring and Symptom Management	- Wearable Devices and Mobile Apps: collecting real-time data on physical activity, sleep quality, heart rate, and other relevant parameters to monitor disease activity. - Flare-Up Analysis: using algorithms to detect flare-ups or significant changes in disease activity, allowing for timely interventions.	- Integration with Internet of Things (IoT): developing intelligent and autonomous monitoring systems that integrate data from various devices and sensors. - Advanced Telemedicine: implementing AI-enhanced telemedicine platforms for more effective remote patient management, improving care quality and reducing hospitalizations. - Proactive Interventions: using AI to predict and prevent disease exacerbations through personalized and timely interventions.
Research and Development Support	- Big Clinical and Genomic Data Analysis: utilizing machine learning algorithms to identify new therapeutic targets and biomarkers. - Intelligent Database Creation: developing AI-supported databases that facilitate research and innovation in rheumatology.	- Standardized Global Databases: creating shared, standardized repositories globally to improve data access and accelerate the development of new therapies, ensuring comprehensive representation of all patient groups, including, for example, pediatric populations. - Clinical Review Automation: Developing AI platforms to automatically analyze clinical study results, optimizing the review and publication process. - Interdisciplinary Collaborations: Promoting synergies between researchers, clinicians, and AI developers to continuously innovate research methodologies in rheumatology.

## Data Availability

We have not generated new data.
